# Editorial

**Published:** 1885-03

**Authors:** 


### Editorial.

THE commencement of the Buffalo Medical College took place Tuesday evening, Feb. 24th, at Concert Hall. The Rev. S. R. Fuller, Rector of St. John's Church, this city, gave the address to the graduating class, and Mr. James Frazer Gluck to the Alumni. The following are the names of the new graduates:

1. Frank Fowler Dow, Peru, Ind.
2. Edward Ledgyard Gager, Buffalo, N. Y.
3. Helen Phelps Morehouse, Buffalo, N. Y.
4. Charles Kennedy, Buffalo, N. Y.

5. Benjamin Willis Cornwell, Alden, N. Y.
6. James F. Sherman, Rush, N. Y.
7. Lewis B. Andrews, Bergen, N. Y.
8. Pine Elijah Bush, Richfield Springs, N. Y.
9. Walden Manley Ward, Perrysburg, N. Y.
10. James W. Chace, Corry, Pa.
11. Frank Theo. Noeson, Sinclairville, N. Y.
12. Daniel H. Brennan, Albion, N. Y.
13. Sarah E. Simonet, Craghan, N. Y.
14. M. Jean Wilson, Wethersfield, N. Y.
15. Wm. Eaton Robbins, N. Evans, N. Y.
16. Thomas Francis Dwyer, Buffalo, N. Y.
17. Asbury H. Baker, Dundee, N. Y.
18. James Christopher Wheeler, Dunkirk, N. Y.
19. Jacob Goldberg, Buffalo, N. Y.
20. Charles S. Logan, Arnot, Pa.
21. Francis Strickland Comfort, Campden, Ont.
22 Melvin Byron Huff, Lyons, N. Y.
23. Sewell A. Brooks, Colden, N. Y.
24. Clarence King, Machias, N. Y.
25. James F. Crowley, Batavia, N. Y.
26. Edmund Townsend, Bergen, N. Y.
27. Edgar J. Foote, Lockport, N. Y.
28. William W. Hall, Morris, N. Y.
29. Wm. J. Coyle, Binghamton, N. Y.
30. Wm. O. A. Langs, Suspension Bridge N. Y.
31. Horace Broson Gee, Newark, N. Y.
32. Geo. Webber Cutter, Buffalo, N. Y.
33. Stephen J. Spencer, Ellicottville, N. Y.
34. Bloom Warren Ganoung, Lincoln, Neb.
35. Wm. Spence Gillmor, N. Hector, N. Y.
36. John Ketchum, Santa Barbara, Cal.
37. Edward J. Hobday, Buffalo, N. Y.
38. Francis D. Proctor, Portville, N. Y.
39. John Sidney Champlin, Buffalo, N. Y.
40. Ferdinand Fromholzer, Ruhmannsfelden, Germany.
41. C. Dobyn Curry, A. B., M. D., Minden. Ont.
42. Charles James Carrick, Portageville, N. Y.
43. Theo. C. Greene, Hornellsville, N. Y.
44. George E. Alexander.
45. George W. McClellan, Alton, Ont.
46. Charles M. Walrath, Franklinville, N. Y.

NOTE.—The name of Mathias Schmitz is to be added to the above list of graduates. He passed last year, but was under age.

During the previous day the Alumni of the College met. Able papers were read by Dr. W. W. Potter, Dr. E. C. W. O'Brian, Dr. W. D. Granger and Dr. C. S. Van Pelt, and the following officers elected for the ensuing year:

President—Dr. F. E. Dewey, Peterboro, N. Y.

First Vice-President—C. Diehl.

Second Vice-President—D. W. Harrington.

Third Vice-President—Mary Berkes.

Fourth Vice-President—Charles Van Pelt, Detroit, Mich.

Fifth Vice-President—W. J. French, West Valley, N. Y.

Secretary—John J. Walsh, Buffalo.

Trustees—C. C. Wyckoff, W. C. Phelps, P. W. Van Peyma, Henry Lapp, E. C. W. O'Brian.

Executive Committee—Charles A. Ring, Frank H. Potter, James W. Putnam; *ex-officio* members, Charles Cary, F. E. Dewey.

The following names were recommended for honorary membership: James Frazer Gluck, Buffalo; Dr. A. Dagenais, Buffalo; Dr. W. D. Granger, Buffalo; Dr. P. H. Strong, Buffalo; Dr. Henry Carpenter, Oneida; William Taylor, Canastota; C. C. Griffin, Vinton, Ia.; T. M. Culver, Urbana, O.; J. C. Green, Buffalo.

